# Pneumothorax in patients with idiopathic pulmonary fibrosis: a real-world experience

**DOI:** 10.1186/s12890-020-01370-w

**Published:** 2021-01-06

**Authors:** Ryo Yamazaki, Osamu Nishiyama, Kyuya Gose, Sho Saeki, Hiroyuki Sano, Takashi Iwanaga, Yuji Tohda

**Affiliations:** Department of Respiratory Medicine and Allergology, Kindai University Faculty of Medicine, Osakasayama, Osaka Japan

**Keywords:** Idiopathic pulmonary fibrosis, Hospitalization, Pneumothorax, Recurrence, Treatment

## Abstract

**Background:**

Some patients with idiopathic pulmonary fibrosis (IPF) develop pneumothorax. However, the characteristics of pneumothorax in patients with IPF have not been elucidated. The purpose of this study was to clarify the clinical course, actual management, and treatment outcomes of pneumothorax in patients with IPF.

**Methods:**

Consecutive patients with IPF who were admitted for pneumothorax between January 2008 and December 2018 were included. The success rates of treatment for pneumothorax, hospital mortality, and recurrence rate after discharge were examined.

**Results:**

During the study period, 36 patients with IPF were admitted with pneumothorax a total of 58 times. During the first admission, 15 patients (41.7%) did not receive chest tube drainage, but 21 (58.3%) did. Of the 21 patients, 8 (38.1%) received additional therapy after chest drainage. The respective treatment success rates were 86.6% and 66.7% in patients who underwent observation only vs chest tube drainage. The respective hospital mortality rates were 13.3% and 38.0%. The total pneumothorax recurrence rate after hospital discharge was 34.6% (n = 9).

**Conclusions:**

Pneumothorax in patients with IPF was difficult to treat successfully, had a relatively poor prognosis, and showed a high recurrence rate.

## Background

Idiopathic pulmonary fibrosis (IPF) is a specific form of chronic, progressive and fibrosing lung disease of unknown etiology [1]. The prognosis for patients with IPF is poor, and the median survival is 3–5 years. However, its natural course is highly variable [2, 3]. Many patients with IPF undergo acute respiratory events such as acute exacerbation (AE), pulmonary infection, pulmonary edema due to heart failure, pulmonary embolism, mediastinal emphysema, and pneumothorax [3–6]. The rates of pneumothorax reportedly range from 2 to 20% in patients with IPF, which is second highest to the rates occurring in patients with chronic obstructive pulmonary disease (COPD) [7–11]. It has been shown that pneumothorax was significantly associated with poor survival in patients with IPF [11].

In clinical practice, pneumothorax associated with IPF shows a wide range of severity, from mild, involving a small area that does not require any type of treatment, to severe, which is refractory to intervention. The characteristics of pneumothorax in patients with IPF have not been elucidated. This study aimed to clarify the clinical course, actual management, and treatment outcome of pneumothorax in patients with IPF.

## Methods

### Patients

We retrospectively reviewed the clinical records of all patients with IPF who were first admitted for pneumothorax to the Kindai University Hospital from January 2008 to December 2019. The diagnosis of IPF was made according to the classification used in the INPULSIS trial [12]. Briefly, patients who had histological evidence of a usual interstitial pneumonia pattern on a surgical lung biopsy specimen were included. In the absence of a biopsy specimen, patients with the following findings on high-resolution computed tomography (HRCT) were diagnosed with IPF: honeycombing and/or the presence of reticular abnormality and traction bronchiectasis without features suggestive of alternative causes. Patients with concomitant pleuroparenchymal fibroelastosis (PPFE) were excluded.

### Data collection

We recorded the clinical characteristics of all the study patients, which included the following: findings on physical examination, standard laboratory tests performed on admission, use of long-term oxygen therapy (LTOT), and treatments for IPF. Pulmonary function tests performed within 1 year prior to admission were used for assessment of baseline pulmonary function. We also assessed the specific treatments for pneumothorax.

### Successful treatment for pneumothorax

Treatments for pneumothorax were evaluated with regard to whether or not they were successful. Successful management of pneumothorax was defined as hospital discharge without a chest tube. A successful chest tube treatment was defined as removal of the chest tube.

### Survival assessment

In-hospital deaths and deaths after discharges were retrospectively obtained from the study patients’ hospital charts.

### Recurrence of pneumothorax after first discharge from the hospital for successful treatment of first pneumothorax

Data on recurrence of pneumothorax was assessed. The time to recurrence was defined as the number of days from the date of discharge from the first pneumothorax until the date of admission for recurrence of pneumothorax. Contralateral pneumothorax was not included in the recurrence.

### Statistical analysis

Continuous variables were expressed as means ± standard deviation (SD). Categorical variables were expressed as frequencies. We performed logistic regression analysis to determine predictors of hospital death. *P* values less than 0.05 were considered statistically significant. The median duration of survival after discharge was estimated on a Kaplan–Meier survival curve. Statflex ver.6 software (Artech, CO., Ltd., Osaka, Japan) was used for analysis.

## Results

During the survey period, 36 patients with IPF were admitted to our university hospital for pneumothorax a total of 58 times. The characteristics of the patients before their first admission are shown in Table 1. This study included 29 men and 7 women. The mean age of the study patients was 75.5 ± 5.4 years, and the mean body mass index was 18.5 ± 4.1 kg/m^2^. The mean forced vital capacity (FVC) was 55.8% ± 16.5% predicted and the mean diffusing capacity for carbon monoxide (DLco) was 62.4% ± 14.9% predicted. Twenty-six patients (72.2%) had a smoking history. Seven patients (19.4%) were receiving corticosteroids for IPF.

**Table 1:** Baseline characteristics of the study patients and treatments for IPF before the first admission for pneumothorax

Characteristics	N = 36
Age, year	75.5 ± 5.4
Gender	
Male/female	29/7
Body mass index^a^, kg/m^2^	18.5 ± 4.1
Preadmission of PFT	
FVC^b^, L	1.6 ± 0.6
FVC^b^, % predicted	55.8 ± 16.5
FEV_1_^b^, L	1.5 ± 0.5
FEV_1_^b^, % predicted	66.2 ± 18.4
FEV_1_/FVC^b^, %	91.7 ± 10.6
DLco^c^, mL/min/mmHg	8.3 ± 2.2
DLco^c^, % predicted	62.4 ± 14.9
Smoking status	
Current/former/never	1/25/10
Treatment for IPF at baseline	
Pirfenidone	4
Nintedanib	3
Corticosteroid	7
Cyclosporine	4
None	22
Long-term oxygen therapy	
Yes/no	9/27

Values are shown as actual numbers or means ± standard deviation

N = 36 (^a^N = 35, ^b^N = 27, ^c^N = 12)

DLco, diffusing capacity for carbon monoxide; FEV_1_, forced expiratory volume in 1 s; FVC, forced vital capacity; IPF, idiopathic pulmonary fibrosis; PFT, pulmonary function tests

The clinical data of study patients at the first admission are shown in Table 2. The mean values for Krebs von der Lungen-6 (KL-6), partial pressure of arterial oxygen/fraction of inspiratory oxygen (PaO_2_/FiO_2_), and partial pressure of arterial carbon dioxide (PaCO_2_) were 957 ± 744 U/mL, 295 ± 100, and 46.8 ± 9.8 mm Hg, respectively.

**Table 2:** Clinical data at the first admission for pneumothorax

Variable	N = 36
KL-6^a^, U/mL	972 ± 744
Arterial blood gas	
pH^b^	7.39 ± 0.06
PaO_2_/FiO_2_ ratio^b^	295 ± 100
PaCO_2_^b^, mmHg	46.8 ± 9.8

Values are shown as actual numbers or means ± standard deviation

N = 36 (^a^N = 28, ^b^N = 33)

KL-6, Krebs von der Lungen-6; PaCO_2_, partial pressure of arterial carbon dioxide; PaO_2_/FiO_2_, partial pressure of arterial oxygen/fraction of inspiratory oxygen

The success rates of each treatment for pneumothorax administered during the first admission and the success rates of each treatment administered during the first combined with all of the subsequent admissions of the study patients for recurrent pneumothorax are shown in Table 3. During the first admission, 15 patients (41.7%) did not undergo and 21 (58.3%) underwent chest tube drainage. Of the 21 patients undergoing drainage, 8 (38.1%) received additional therapy after drainage. The additional therapies included placement of an endobronchial Watanabe spigot (EWS) and pleurodesis with fibrin glue and/or a blood patch. The respective rates of successful treatment after the first admission were 86.6% and 66.7% in patients who underwent observation only or underwent chest tube drainage. The respective rates of success after the first admission of chest tube drainage plus the additional therapy of EWS placement or plus pleurodesis (n = 2) after the first admission were 57.1% (n = 7) and 50.0% (n = 2). The total success rate of each treatment administered during all of the admissions was similar to the success rate of each treatment during the first admission only. In patients whose chest tube could be removed, the mean lengths of time until removal for the first admission and total number of admissions were 20.7 ± 14.0 and 23.6 ± 18.5 days, respectively. Some patients remained hospitalized even after the chest tube was removed, mainly because of subsequent type 2 respiratory failure and/or pulmonary infection.

**Table 3:** Rates of successful treatment for pneumothorax without surgical intervention

Treatment	First admission		Total admission	
	Number of patients	Number of patients successfully treated (%)	Number of patients	Number of patients successfully treated (%)
All patients	36	27 (75.0)	58	49 (84.4)
Observation only	15	13 (86.6)	29	26 (89.6)
Chest tube	21	14 (66.7)	29	17 (58.6)
Chest tube only	13	9 (69.2)	15	10 (66.6)
Chest tube plus additional therapy	8*	5 (62.5)	12*	7 (58.3)
EWS placement	7	4 (57.1)	10	5 (50.0)
Pleurodesis with fibrin glue and/or blood patch	2	1 (50.0)	3	2 (66.6)

Two patients during the first admission and 4 during all admissions combined underwent surgery after chest tube placement and some additional therapies resulting in chest tube removal

^*^One patient underwent EWS followed by pleurodesis

EWS, endoscopic Watanabe spigot

The hospital mortality rates for the first admission and for all the admissions combined were 27.7% and 24.1%, respectively (Table 4). The mean durations of hospitalization for the first and total number of admissions were 34.2 ± 27.5 days and 31.0 ± 28.5 days, respectively. Hospital mortality rates were higher in patients who underwent chest tube drainage than in patients who did not undergo drainage during both the first admission and during the total number of admissions, although the differences were not significant.

**Table 4:** Hospital mortality according to treatments for pneumothorax and success of chest tube removal

	First admission		Total admission	
	Number of patients	Hospital death (%)	Number of patients	Hospital death (%)
All patients	36	10 (27.7)	58	14 (24.1)
Observation only	15	2 (13.3)	29	3 (10.3)
Chest tube	21	8 (38.0)	29	11 (37.9)
Successful chest tube removal	14	3 (21.4)	17	3 (17.6)
Failure of chest tube removal	5	5 (100)	8	8 (100)
Removed with surgery	2	0 (0)	4	0 (0)

The recurrence rates of pneumothorax in patients who were discharged alive are shown in Table 5. Pneumothorax recurred in 9 of 26 (34.6%) patients discharged alive after the first admission. Among the 9 patients, 8 patients (8.8%) recurred within 1 year. Differences between recurrence rates after each treatment were not significant. However, the recurrence rates tended to be higher in patients who had undergone chest drainage plus additional therapy.

**Table 5:** Relationships between treatment for pneumothorax and recurrence in patients who were discharged alive

Treatment for the pneumothorax	First admission		Total admissions	
	Number of patients	Number of patients recurring (%)	Number of patients	Number of patients recurring (%)
All patients	26*	9 (34.6)^†^	44*	14 (31.8)^†^
Observation only	13	5 (38.4)	26	10 (38.4)
Chest tube	13	4 (30.7)	18	4 (22.2)
Chest tube only	7	2 (28.5)	9	2 (22.2)
Chest tube plus additional therapy	4^‡^	2 (50.0)	5^§^	2 (40.0)
Surgery**	2	0 (0)	4	0 (0)

The last treatments in the admission were listed

EWS, endoscopic Watanabe spigot

^*^Including 2 patients with bilateral pneumothorax

^**^One patient (50%) recurred among the patients who underwent surgery during the first admission. One patient recurred (25.0%) among the patients who underwent surgery during all the admissions combined

^†^Including 1 patient with bilateral pneumothorax

^‡^Three patients underwent EWS after chest tube drainage. One patient underwent blood patch pleurodesis and fibrin glue pleurodesis after chest tube drainage

^§^Four patients underwent EWS after chest tube drainage. One patient underwent blood patch pleurodesis and fibrin glue pleurodesis after chest tube drainage

None of the variables assessed at admission and none of the types of treatments were identified as significant predictors of hospital mortality. The estimated median duration of survival was 154 days after discharge of the first admission among patients who could discharge alive.

## Discussion

Pneumothorax can occur in patients with underlying lung diseases; its rate of frequency is highest in patients with COPD and second-highest in those with interstitial pneumonia [10]. Pneumothorax that occurs in patients with chronic lung disease is reportedly associated with higher mortality and recurrence rates than primary spontaneous pneumothorax that occurs in patients without chronic lung disease [9, 10, 13]. Our study shows that pneumothorax in patients with IPF was difficult to treat successfully, shows a high recurrence rate, and has a relatively poor prognosis.

The insertion of a chest tube for the initial management of secondary pneumothorax warrants discussion. In our study, some patients did not undergo chest tube drainage. The British Thoracic Society (BTS) recommends insertion of a chest tube when the depth of the pneumothorax from the chest wall is > 2 cm and/or when the patient shows signs and symptoms of pneumothorax [14]. When the depth is ≤ 2 cm, temporal aspiration or observation with oxygen inhalation are recommended. In our hospital, we generally followed the recommendations of the BTS, although insertion of a chest tube was ultimately performed based on the treating physician’s judgement.

The BTS recommends surgery for patients with a persistent air leak from the chest tube placed for drainage [14]. However, some patients with secondary spontaneous pneumothorax are not candidates for surgical intervention because of age, poor pulmonary function, or other comorbidities. In addition, acute exacerbation of IPF can occur after surgery [15]. Actually, only 2 patients during the first admission and 4 during all the admissions combined underwent surgery after chest tube drainage, plus additional treatments. Successful thoracoscopic surgery for intractable pneumothorax under local and epidural anesthesia was reported [16]. V-V ECMO utilization for such a difficult case was also reported [17]. However, no patients underwent surgery with such strategies in the present study.

Chemical pleurodesis can be a nonsurgical option for patients with persistent air leakage from the chest tube [14]. Complete re-expansion of the lung is needed to achieve successful chemical pleurodesis. However, some IPF patients with pneumothorax do not obtain re-expansion of the lung because of the distinctive rigidity of the lung parenchyma in IPF [18]. Another concern with chemical pleurodesis is the risk of restrictive pulmonary dysfunction [19] and acute exacerbation of IPF after the procedure [20]. Probably for these reasons, chemical pleurodesis was not performed for any of the study patients. As another nonsurgical option, pleurodesis performed with an autologous-blood patch or fibrin glue has been reported to be effective [21, 22]. Although adequate pulmonary re-expansion is needed to achieve pleurodesis, the risks of restrictive pulmonary dysfunction and acute exacerbation of IPF after these procedures might be lower than they are after chemical pleurodesis. In our patient series, a respective 2 patients during the first admission and 3 patients during all the admissions combined underwent pleurodesis by fibrin glue or autologous blood patch for success rates of 50% and 66%.

An EWS is a silicone spigot used to occlude a bronchus [23]. The EWS has been reported to be effective for reducing air leakage in about 50% of patients [24]. In our study, the EWS showed relatively high success rates of 57.1% and 50.0% for the first and all the admissions combined, respectively. Given that the EWS has been found to be useful even in patients who have incomplete re-expansion of the lung, placement of an EWS might be suitable for patients with IPF and pneumothorax with persistent air leakage from the chest tube. However, placement of the EWS requires sedation and intubation of the patient. Poor lung function and/or low oxygenation might indicate that a patient would not tolerate the procedure. Criteria for safely performing placement of an EWS need to be established.

Hospital mortality rates of patients with secondary spontaneous pneumothorax have been reported to range from 4.5% to 17.9% [10, 25]. In these reports, the most frequent underlying disease was pulmonary emphysema. By contrast, a higher hospital mortality rate has been reported for pneumothorax in patients with IPF [6]. The hospital mortality rates in our study ranged from 24 to 28%, which were relatively high, although similar to the previous report [6]. The mortality of patients who required chest tube drainage was even higher in our study.

The recurrence rate of secondary spontaneous pneumothorax varies according to the published literature, ranging from 30 to 80% [14, 25, 26]. The recurrence rate of pneumothorax in patients with IPF is reportedly high (70.6%) [11]. In our study, 34.6% of patients with IPF developed recurrence after their first admission and subsequent discharge for pneumothorax. Furthermore, the majority of recurrences were observed within the first year after discharge. Given that recurrence of pneumothorax in a patient with IPF confers additional risk of death, preventative methods for recurrence should be developed.

This study has limitations. First, it was a single-center retrospective study of a relatively small number of patients. Second, the treatment for pneumothorax was not uniform because it was performed by individual physicians and based on their individual judgements. Third, the study might have included patients with PPFE [27, 28]. Although major efforts were made to exclude patients with PPFE based on the chest HRCT, some patients with subclinical PPFE could have been included. Histological PPFE seen in the upper pulmonary lobes was reported to trigger pneumothorax in patients with IPF [29]. Further studies of pneumothorax in IPF patients should preferably base study enrollment on a histological evaluation of a lung specimen.

To the best of our knowledge, the study is the first to evaluate additional treatments in IPF patients with pneumothorax and persistent air leakage after placement of a chest drain. The efficacy of combined therapy, consisting of treatments in addition to chest tube drainage should be improved.

## Conclusions

Pneumothorax in patients with IPF is difficult to treat successfully, and has a relatively poor prognosis and high recurrence rate.

## Data Availability

The datasets used and/or analyzed during the current study are available from the corresponding author upon reasonable request.

## Abbreviations

AE: Acute exacerbation
COPD: Chronic obstructive pulmonary disease
DLco: Diffusing capacity for carbon monoxide
EWS: Endobronchial Watanabe spigot
HRCT: High-resolution computed tomography
IPF: Idiopathic pulmonary fibrosis
KL-6: Krebs von der Lungen-6
LTOT: Long-term oxygen therapy
PaCO_2_: Partial pressure of arterial carbon dioxide
PaO_2_/FiO_2_: Partial pressure of arterial oxygen/fraction of inspiratory oxygen
PPFE: Pleuroparenchymal fibroelastosis
